# WiFi Signal-Based Gesture Recognition Using Federated Parameter-Matched Aggregation

**DOI:** 10.3390/s22062349

**Published:** 2022-03-18

**Authors:** Weidong Zhang, Zexing Wang, Xuangou Wu

**Affiliations:** 1 School of Computer Science and Technology, Anhui University of Technology, Maanshan 243032, China; weiddzhang@163.com (W.Z.); zzexingwang@126.com (Z.W.); 2 Anhui Engineering Laboratory for Intelligent Applications and Security of Industrial Internet, Maanshan 243032, China

**Keywords:** IoT, federated learning, gesture recognition, CSI

## Abstract

Gesture recognition plays an important role in smart homes, such as human–computer interaction, identity authentication, etc. Most of the existing WiFi signal-based approaches exploit a large number of channel state information (CSI) datasets to train a gestures classification model; however, these models require a large number of human participants to train, and are not robust to the recognition environment. To address this problem, we propose a WiFi signal-based gesture recognition system with matched averaging federated learning (WiMA). Since there are differences in the distribution of WiFi signal changes caused by the same gesture in different environments, the traditional federated parameter average algorithm seriously affects the recognition accuracy of the model. In WiMA, we exploit the neuron arrangement invariance of neural networks in parameter aggregation, which can improve the robustness of the gesture recognition model with heterogeneous CSI data of different training environments. We carried out experiments with seven participant users in a distributed gesture recognition environment. Experimental results show that the average accuracy of our proposed system is up to 90.4%, which is very close to the accuracy of state-of-the-art approaches with centralized training models.

## 1. Introduction

With the development of the respective technologies of the intelligent Internet of Things, gesture recognition is attracting more and more attention in smart homes, such as human–computer interaction, identity authentication, etc. Traditional gesture recognition approaches include computer vision-based technology [1,2,3], wearable device-based technology [4,5,6], and so on. Although these approaches can realize gesture recognition with high efficiency and low delay, they usually require special equipment, which is either expensive or inconvenient to wear.

Recently, WiFi-based gesture sensing has been of wide interest, because it has the advantages of low cost and easy deployment. Most of the existing approaches exploit feature extraction from the channel state information (CSI) of WiFi signals and build a gesture recognition model. For example, Mohammed et al. proposed a device-free WiFi-based gesture recognition system [7], which can extract the duration of the gesture from the CSI fluctuations generated by hand motion to recognize different gestures. TW (see [8]) removes noise from CSI by principal component analysis (PCA), and performs gesture recognition by building a CNN model. Although these methods can perform gesture recognition by CSI, none of them is environment robust, and the prediction accuracy will be greatly reduced if a new user is predicted in a new environment. Zhang et al. proposed Widar3.0, a WiFi-based zero-effort cross-domain gesture recognition system [9] which establishes an environment-independent feature body-coordinate velocity profile (BVP).

Due to differences in user behavior and the unbalanced distribution of user characteristics, the accuracy of the model can only be guaranteed when a sufficient number of users are involved in training. Furthermore, in a real Internet of Things (IoT) environment, it is difficult to obtain enough user data to train a centralized model due to privacy and transmission costs. Federated learning trains distributed models by collecting model parameters from numerous data providers, which can alleviate the problems of insufficient data and data privacy [10]. However, there are differences in the distribution of WiFi signal changes caused by the same gesture in different environments, and the traditional federated parameter average algorithm seriously affects the recognition accuracy of the model [11].

To address the above problem, we propose WiMA, a federated learning-based gesture recognition framework with WiFi signals. In WiMA, we train the BVP-based gesture recognition model on the federated learning clients, using the permutation invariance of the neural network to match neurons with similar feature extraction functions when the server aggregates the parameters, and freeze the matched neurons in layers when clients update the parameters. This allows a more comprehensive extraction of BVP features of the same gesture for different users, thus improving the robustness of the model to unbalanced data.

The main contributions of this paper can be summarized as follows:

- We propose cross-local gesture recognition based on matched average federation learning, aiming to solve the problem of low accuracy of WiFi gesture recognition due to limited user samples and distribution differentiation.
- To realize robust cross-environment gesture recognition, we use BVP and construct a deep learning model to build a local model, and then fuse the parameters between the local models by federated average algorithm, and use the fused parameters to replace the local modeling parameters.
- We carried out experiments with seven participant users in a distributed gesture recognition environment. Experimental results show that the average accuracy of our proposed system is up to 90.4%, which is very close to the accuracy of state-of-the-art approaches with centralized training models.

The rest of this paper is organized as follows: Section 1 briefly summarizes the overall work; Section 2 details the current work related to WiFi gesture recognition; Section 3 describes the basic techniques; Section 4 explains the motivation for using the federated parameter matched averaging algorithm; Section 5 details the WiFi signal-based gesture recognition system with matched averaging federated learning; and the performance of the proposed algorithm is verified in Section 6.

## 2. Related Work

In recent years, with the combination of IoT and AI, model-based indoor WiFi action recognition has started to emerge. However, the data required for training models are often private, and federation learning has emerged to provide data protection for distributed model training. This section focuses on recent research related to WiFi action recognition and federation learning.

### 2.1. WiFi Gesture Recognition

In [12], Ding et al. proposed a passive device-free fall detection system, based on WiFi framework for smart homes, which collects disturbance signals caused by human motion from smart homes, performs a discrete wavelet transform on the data to eliminate random noise, and then uses it as an input to a recurrent neural network to identify fall states. In [13], Palipana et al. proposed FallDeFi, which use the traditional short-time Fourier transform (STFT) to extract the time–frequency features in the WiFi signal, and the features that are resilient to environmental changes are selected by a sequential forward selection algorithm with a high fall detection rate.

In [14], Venkatnarayan et al. proposed a WiFi-based multi-user gesture recognition method (MiMu), which automatically determines the number of gestures to be performed simultaneously, generates virtual samples from a training sample of individual users, and recognizes gestures from comparisons with virtual samples. In [15], Golestani et al. proposed a wireless system for human activity recognition based on magnetic induction, combined with machine learning techniques to detect a wide range of human motion.

In [7], Al-qaness et al. proposed a WiFi-based device-free gesture recognition system (WiGeR), which obtained the CSI fluctuation trend generated by hand motion by filtering out the noise using the fluctuation of channel state information (CSI) of WiFi signal caused by hand motion. In [16], Shang et al. proposed a sign language recognition system (WiSign) based on WiFi signals, which extracts the multi-path distortion fluctuations caused by different hands and arms in WiFi signals from CSI. In [8], Wu et al. proposed an opposite robust PCA (OR-PCA) approach, which can obtaine correlations between human activities and their resulting changes in channel state information values, thus eliminating the influence of the background environment on correlation extraction. In [17], Li et al. proposed WiHF, which derives a domain-independent motion change pattern of arm gestures from WiFi signals, rendering the unique gesture characteristics and the personalized user performing styles. In [18], Gu et al. proposed a gesture recognition system based on the channel attention mechanism and CNN-LSTM fusion model, which uses CNN-LSTM to extract spatiotemporal features with the help of attention mechanisms. In [19], Tang et al. proposed a one-dimensional parallel long short-term memory–fully convolutional network (LSTM-FCN), which uses LSTM to extract temporal features in user gesture recognition data, and FCN to extract spatial features of data to jointly solve the task of user gesture recognition from two dimensions.

However, these methods for action recognition either require the use of expensive equipment or are supported by large amounts of experimental data, and can only recognize a small number of user actions or gestures.

### 2.2. Federation Learning

Federated learning was first proposed by McMahan et al. [10], where a server extracts the parameters of a multi-user local machine learning model, and the data can be trained collaboratively on a distributed model without leaving the local area; it can protect sensitive user information. In [20], S et al. proposed an adaptive update algorithm for federation learning model parameters, which solves for the optimal number of client updates by comparing the training accuracy of centralized learning, analyzing the model convergence bound, and relating the number of client local updates to the model accuracy, combined with the constraints on the client resource consumption and the total number of communication rounds. However, traditional federation learning algorithms only weight the model parameters uploaded by different clients and do not take into account the replacement invariance of each neuron in the model, which often degrades the performance for scenarios with unbalanced data distribution.

In [21], Wang et al. proposed the federated matching average algorithm (FedMA), which constructs a shared global model by layer matchingand averaging the extracted hidden elements with similar features. It can extract the data distribution difference characteristics of different clients to deeply match the calculation units of different client models, which can alleviate the problem of accuracy degradation caused by data distribution differences. In [22], Tang et al. proposed a federated matched averaging algorithm with information-gain-based sampling, which calculates the information gain of the parameters before transmitting the data, reducing the number of parameters sent by the client through the sampling algorithm.

Federated learning can well solve the privacy problem of multi-user data, and the matching average algorithm can alleviate the heterogeneity problem of multi-user data.

## 3. Background

### 3.1. CSI and BVP

In frequency division multiplexing (OFDM) systems, by using current commercial Wi-Fi equipment, *S* subcarriers represented by complex values can be collected from each packet. CSI can be defined as

(1) $$H\left(f_{k},t\right)=\left|H\left(f_{k},t\right)\right|e^{j∠H(f_{k},t)},k\in \left[1,S\right]$$

where $\left|H(f_{k},t)\right|$ and $∠H(f_{k},t)$ represent the subcarrier $f_{k}$ as the center frequency and the *t*th timestamp CSI values of the amplitude and phase, respectively.

The relative motion between the transmitter and the receiver causes Doppler frequency shift (DFS) [23]. According to CARM, the root reason that leads to DFS is the change of signal propagation path. The frequency shift which results from the reflected signal generated can be written as

(2) $$f_{D}\left(t\right)=-\frac{1}{\lambda }\frac{d}{dt}d\left(t\right)=-f\frac{d}{dt}\tau \left(t\right),$$

where lambda, *f*, and tau(t) correspond to the wavelength of the signal, the subcarrier frequency, and the time of flight of the signal, respectively, and d(t) is the distance of the NLOS path.

**Figure 1:**
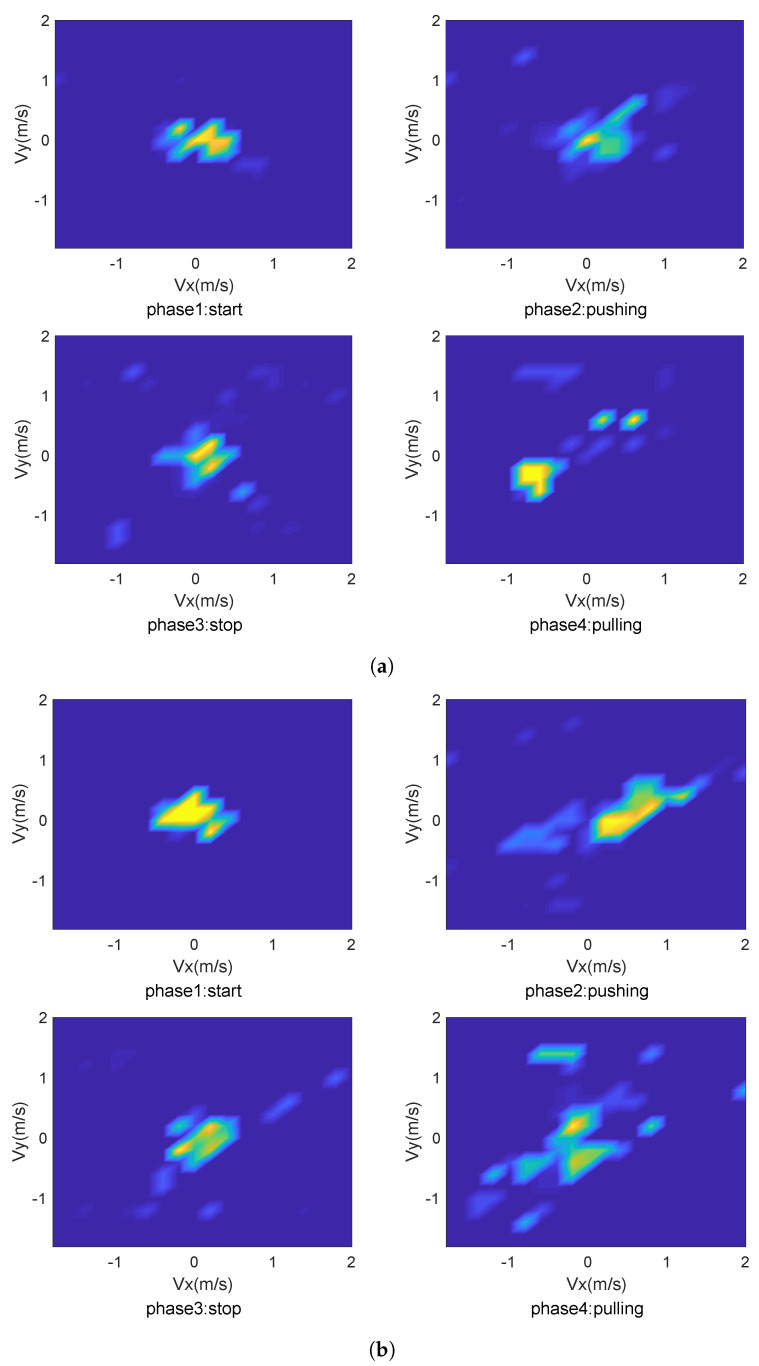
BVP series for different users. (**a**) BVP series of user1. (**b**) BVP series of user2.

When the user performs a gesture, in addition to the motion of various body parts that generate different velocities, these movers also cause relatively non-negligible motion of the DFS. Assuming accumulation caused by Doppler frequency shift of the velocity vector for $\vec{v}$, in each timestamp, note that *k* transceiver link corresponds to the Doppler frequency shift as $F_{D}^{k}\left(\vec{v}\right)$:

(3) $$F_{D}^{k}\left(\vec{v}\right)=c_{x}^{k}\vec{v_{x}}+c_{y}^{k}\vec{v_{y}},$$

where $c_{x}^{k}$ and $c_{y}^{k}$ are determined by the location of the corresponding transceiver link. Derived from $\vec{v}$, $\vec{v_{x}}$ refers to the user’s face orientation, and $\vec{v_{y}}$ refers to the vertical direction [24]. Therefore,$c_{x}^{k}$ and $c_{y}^{k}$ can be used to solve the possible values of $\vec{v_{x}}$ and $\vec{v_{y}}$, calculate $F_{D}^{k}\left(\vec{v}\right)$, solve the optimal solution of $\vec{v_{x}}$, and $\vec{v_{y}}$ with the measurement DFS isolated from the CSI measurement [25]. Body-coordinate velocity profile (BVP) can be represented by $\vec{v_{x}}$ and $\vec{v_{y}}$. Different users perform the same action with different patterns; taking push and pull as an example, as shown in Figure 1, different users at different phases of the same gesture have different power distribution of speed components and different execution duration.

### 3.2. Federated Learning

According to the definition of federated learning [10], assuming that there are *N* clients participating in the shared model training, the training data owned by the *i* client are $D_{i}$. Assuming that *w* is the model weight parameter, the loss function of a single sample j is $f_{j}\left(\cdot \right)$; therefore, the loss function of the *i*th client is calculated as

(4) $$F_{i}\left(w\right)=\frac{\sum_{j\in D_{i}}f_{j}\left(w\right)}{|D_{i}|}$$

Among them, $|D_{i}|$ represents the size of the dataset $D_{i}$. Then, the loss function of the federated sharing model is

(5) $$F\left(w\right)=\frac{\sum_{i=1}^{N}\left|D_{i}\right|F_{i}\left(w\right)}{\left|D\right|}$$

Among them, $\left|D\right|=\sum_{i=1}^{N}\left|D_{i}\right|$, and note that F(w) cannot be directly computed without sharing information among multiple nodes.

**Figure 2:**
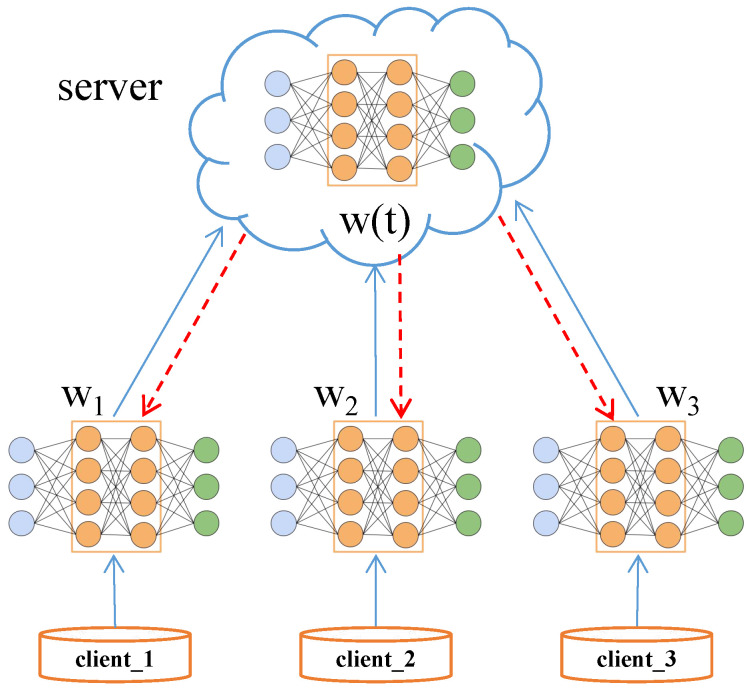
Federal learning framework.

The training process of federated learning is shown in Figure 2. The server collects the model parameters uploaded by each client in each iteration, and then distributes them to each client after weighted averaging to complete the update of local model parameters.

## 4. Analysis and Motivation

In recent years, user gesture recognition technology based on WiFi signals has been widely used in the field of IoT, such as smart homes. Existing gesture recognition methods all require a huge amount of data support. With the help of complex deep learning and other model structures, when the number of participating users in the training set is sufficient, high accuracy can be achieved in the prediction of new users [26,27]. However, in real scenarios, it is difficult to gather enough users to collect enough training data, or the labor and transmission costs of collecting data are higher than the value it can bring. In this context, the recently emerging concept of federated learning may bring new opportunities. Federated learning allows multiple users to collect data locally, and jointly train a common global model by transferring parameters, and without worrying about data transportation costs and privacy leakage.

However, whether federated learning can use data generated by users scattered in different regions to train models with high enough accuracy to predict new users has not yet been verified. To verify the performance of federated learning in gesture recognition application scenarios with different distributions of multi-client user ratios, we carried out the following analytical experiments, and the data and models required for the experiments are detailed in Section 6.

**Figure 3:**
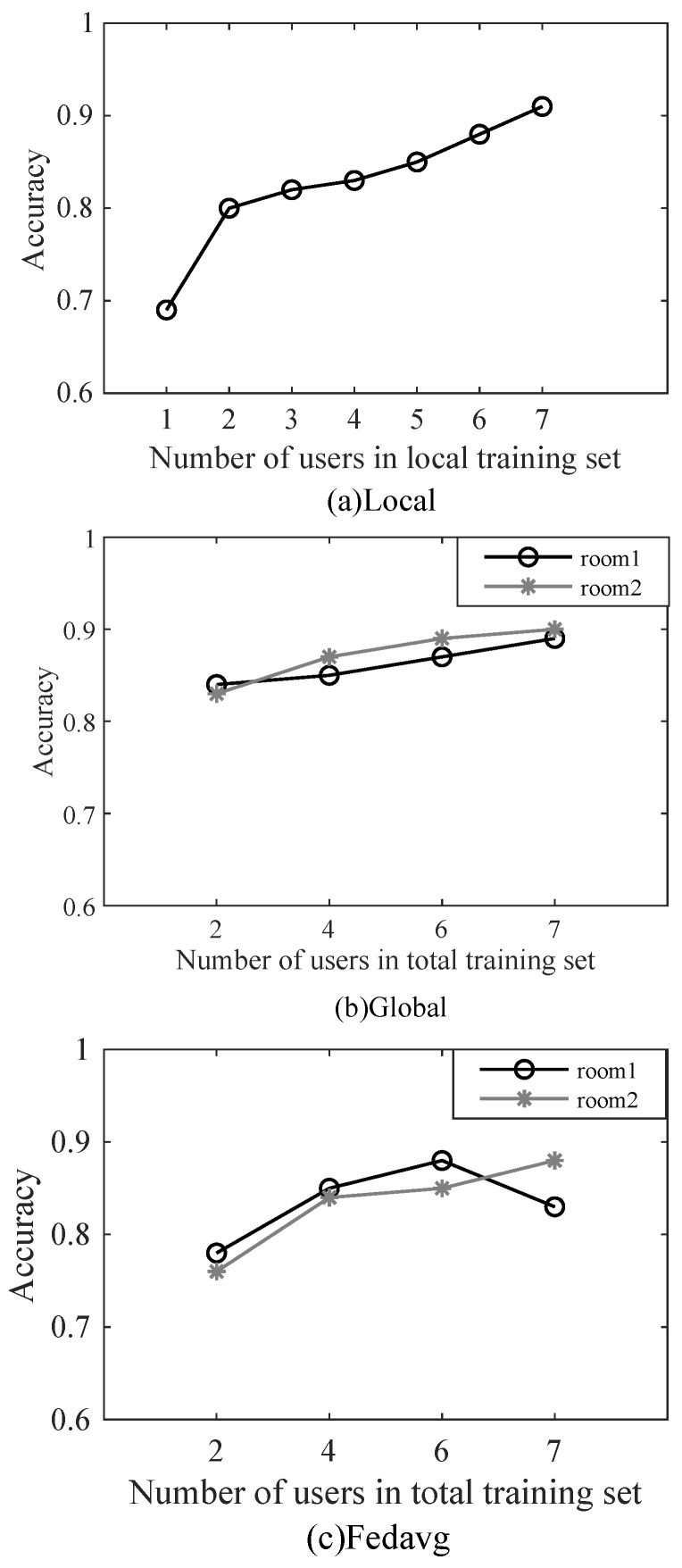
Different method comparison.

We first studied the relationship between the model accuracy and the number of users. The results are shown in Figure 3a. Within a certain range, the model accuracy increases with the increase of the number of users. When the total number of users reaches seven, the model accuracy can exceed 0.9, which can meet the needs of most scenarios. In reality, very few families have seven people.

Then we consider the distributed scenario, disperse the previous seven users into two rooms (two client executables), divide their data into training set and test set first, and then gather them together. This is the difference from the previous experiment, and we then observe the respective test accuracy of the two rooms. The results are shown in Figure 3b. Similar to the single-room centralized scenario, when the total number of users in two rooms reaches seven, their respective model accuracy can reach 0.9.

Finally, we verify our conjecture using FedAvg, a classic algorithm for federated learning. The two rooms each represent a client that learns the model on the local training set, and an additional server is responsible for fusing their model parameters. The result is shown in Figure 3c: as the number of users increases, the accuracy of room2 can exceed 0.9, while the accuracy of room1 decreases after reaching a certain value. According to the description in Reference [21], this is caused by the data heterogeneity in the local data of two room users. This result shows that FedAvg is difficult to adapt to the complex data structure. When the user’s local data are biased, the server-side global model may perform well, while the user’s local model performs differently. The reason is that FedAvg simply weights and averages the local model parameters of each user to achieve overall high accuracy, while ignoring the differences in the characteristics of each local model for its data.

Therefore, we are motivated to use the permutation invariance of neural networks to further search and match the model parameters of individual users by combining neurons with the same feature extractor and encoding the respective differentiated neurons for normalization.

## 5. Design

**Figure 4:**
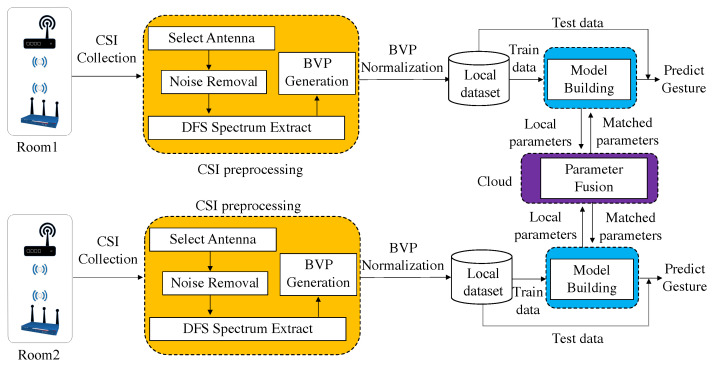
WiMA system architecture.

To train an efficient client federated learning model, we divide the WiMA system into four blocks (as shown in Figure 4), CSI preprocessing block, BVP normalization block, model building block, parameter fusion block, and gesture recognition block.

The CSI preprocessing block extracts DFS from collected raw CSI measurements, and generates BVP from the DFS spectrum. The BVP normalization block is designed to standardize the BVP series data to generate local datasets. The model building block is used to construct local models with training data from local datasets. The parameter fusion block is used to match and fuse the parameters of local models and return match parameters to local models. The gesture recognition block is responsible for distinguishing different user gestures with local models with matched parameters.

### 5.1. CSI Preprocessing

According to IndoTrack [23], the transmitting antenna and the two receiving antennas of the CSI amplitude conjugate multiplication are used to eliminate the quasi-static offset. The band-pass filter is used to filter out-of-band noise, which can remove the random offset. Therefore, in order to preserve non-zero DFS with gaining multipath components, two receiving antennas need to be selected. Widance [28] studied the influence of different antennas on the dynamic path by calculating the variance of CSI amplitudes for different transmit–receive antenna pairs, and the two receive antennas with larger variance in the transmit–receive pair are selected to describe the user-induced dynamic components, which can be used to extract the DFS spectrum and generate the BVP.

### 5.2. BVP Normalization

For the obtained BVP series, we need to normalize the BVP series. Durations of different BVP series samples are not uniform, so it is necessary to upsample, fix the duration of all samples, and normalize all elements in the BVP series. In this way, it can be ensured that the BVP series is only related to user gestures.

### 5.3. Model Building

The clients use different local datasets to train a model with the same structure, and the cloud collects local model parameters for parameter fusion. Each BVP series data can be regarded as a picture sequence, which consists of pictures depicting the distribution of velocity components. Each BVP profile describes the energy distribution of the user performing a certain gesture. We use a convolutional neural network (CNN) as a spatial feature extractor, which can automatically learn parameters and features for complex image problems.

Furthermore, since the BVP series has temporal features, we introduce a recurrent neural network (RNN) to extract such dynamic temporal features. Common models of RNN usually have long short-term memory (LSTM) and gated recurrent unit (GRU). Compared with LSTM, GRU can use fewer parameters and obtain fairly accurate results, so we use GRU to characterize BVP timing.

**Figure 5:**
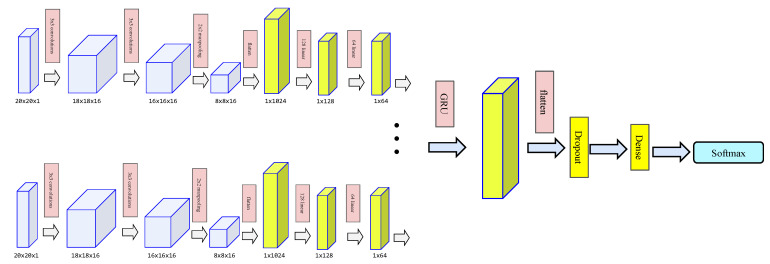
Local model structure.

As shown in Figure 5, the complete network structure of the local model is two 3 × 3 convolutional blocks, a 2 × 2 maximum pooling layer, and two fully connected layers for each BVP profile. The GRU block is then input and the GRU output is expanded, and a dropout layer is introduced to prevent the model from overfitting. Then, the input is extended to the fully connected layer classifier, and, finally, the softmax classifier based on the cross-entropy loss function obtains the prediction result.

### 5.4. Parameter Fusion and Gesture Recognition

For the parameters trained by local models, we propose a federated matching algorithm, whose core is to introduce a permutation matrix to realize the permutation invariance of neurons in the neural network. The simplest single-layer fully connected network can be formulated as $y=\sum_{i=1}^{L}W_{2,i}\sigma \left(<x,W_{1,i}>\right)$, and L is the number of neurons in the hidden layer. Therefore, there are total L! parameter arrangements for $W_{1},W_{2}$. Further,

(6) $$Y=\sigma \left(xW_{1}\right)W_{2}=\sigma \left(xW_{1}\Omega \right)\Omega^{T}W_{2},$$

where Ω is any L×L permutation matrix. For two of the same size datasets, $X_{j},X_{j^{\prime }}$, weight is obtained by training for $W_{1}\Omega_{j},\Omega_{j}^{T}W_{2}$ and $W_{1}\Omega_{j^{\prime }},\Omega_{j^{\prime }}^{T}W_{2}$. Obviously, most likely $W_{1}\Omega_{j}\neq W_{1}\Omega_{j^{\prime }}$ and $\left(W_{1}\Omega_{j}+W_{1}\Omega_{j^{\prime }}\right)/2\neq W_{1}\Omega_{j}$ for any Ω. Therefore, the first thing to restore replacement is $\left(W_{1}\Omega_{j}\Omega_{j^{\prime }}^{T}+W_{1}\Omega_{j^{\prime }}\Omega_{j^{\prime }}^{T}\right)/2\neq W_{1}$. Suppose Wjl is the *l*th neuron learned on dataset $X_{j}$, $\theta_{i}$ represents *i*th neuron in the global model, and c(·) is defined as an appropriate similarity function between a pair of neurons. The permutation optimization problem can be defined as follows:

(7) $$\underset{\tau_{li}^{j}}{min}\sum_{i=1}^{L}\sum_{j,l}\underset{\theta i}{min}\tau_{li}^{j}\cdot c\left(W_{jl},\theta_{i}\right),s.t.\sum_{i}\tau_{li}^{j}=1\forall j,l;\sum_{i}\tau_{li}^{j}=1\forall i,j$$

$\Omega_{\mathrm{jli}}^{T}=\tau_{li}^{j}$ and the weight of a specific provide *j* th local ${W_{j,q},W_{j,2}}_{j=1}^{J}$ provided by *J* local sides, so we calculate the federated neural network weights $W_{1}=\frac{1}{J}\sum_{j}W_{j,1}\Omega_{j}^{T}$ and $W_{2}=\frac{1}{J}\sum_{j}\Omega_{j^{\prime }}^{T}W_{j,2}$.

In order to solve the constraint problem involved in Equation (7), we apply Hungarian matching algorithm and BBPMAP algorithm. This involves a basic concept in the field of deep learning—weight space symmetry—whereby a neural network with multiple latent variables will have multiple local minima, and equivalent models can be obtained by exchanging the positions of the latent variables with each other. According to this symmetry, any given neural network, which differs in many variations only in the order of the parameters, constitutes a practically equivalent local optimum. Since the data for multi-user gesture recognition are often heterogeneous (non-IID), simply averaging the local model parameters for each user as a whole makes it difficult to effectively extract the variability of each user’s local data, thus reducing the accuracy of the user’s local model.

To solve this problem, the server first collects the weights of the first layer from the client, and performs similarity matching and averaging on the neurons in this layer of each client to obtain the first layer weights of the federated model. The server then broadcasts these weights to the client, freezes the parameters of the matched layers, and trains all successive layers in the same way. This process is then repeated to the final layer, where a weighted average is applied to each client’s data based on their class proportions.

## 6. Experiment Results

This section verifies the performance of the proposed algorithm.

### 6.1. Basic Settings

We use the public dataset Widar3.0 [25], which contains 9 gestures by 16 users. We select 6 gestures performed by 12 users in 2 rooms as a dataset. In WiMA, we assume two rooms as two local sides. The user data in each room do not conform to the characteristics of the independent and identical distribution. We randomly select one user locally as the test user, and then randomly select a specified number of users from the remaining users as the local training set. We implement WiMA in MATLAB and Keras.

### 6.2. Benchmark

- Widar3.0: This benchmark is a reproduction of the method in the literature [9], which is a centralized training method, which scrambles all users together to extract BVP data, and divides the training set and test set according to the ratio of 7:3. The network mechanism used is shown in Section 5.3 as well as Figure 5, which is the ideal situation for gesture recognition under the limitation of a fixed number of users and can achieve the highest theoretical accuracy. The final results may differ slightly from the original results.
- Global: The method is to pool the data of seven standard users together and divide the training and validation datasets in the ratio of 7:3.
- FedAvg: The method is to place seven users in two rooms, divide the training and testing data on the datasets of the two rooms, and centralize the two tests on the server-side. The server collects the local models obtained from training on the local data of each room, performs a simple weighted average of the parameters to obtain a new global model, and updates the model for each room.

### 6.3. Experimental Analysis

**Figure 6:**
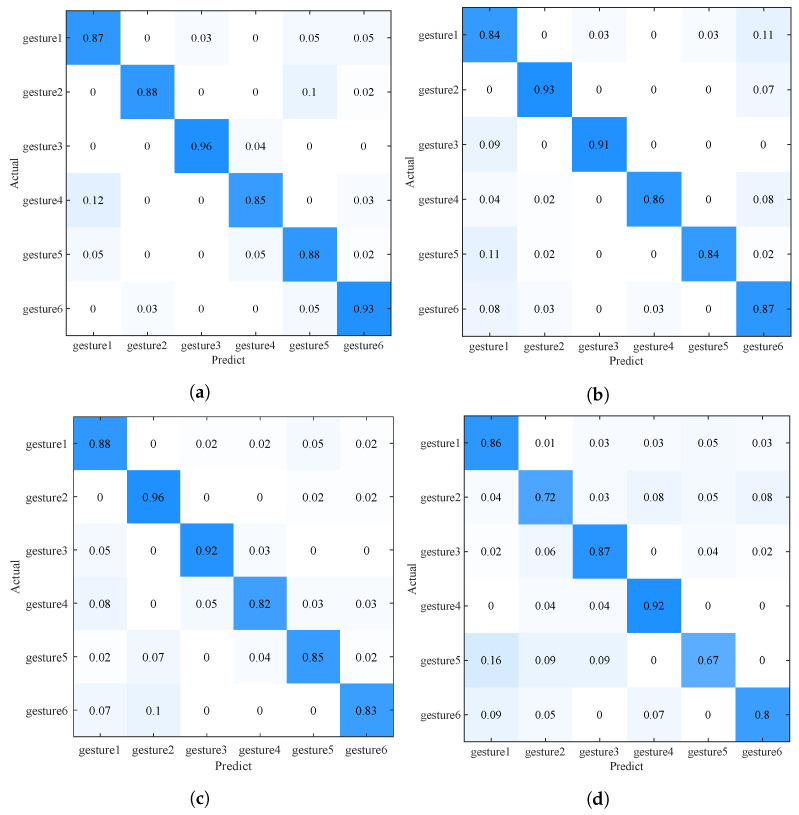
Confusion matrix of WiMA under different number of users. (**a**) Users number = 7, accuracy = 0.90. (**b**) Users number = 6, accuracy = 0.88. (**c**) Users number = 4, accuracy = 0.85. (**d**) Users number = 2, accuracy = 0.79.

Overall accuracy: We tested our method on the server side and saved the result; the result is shown in Figure 6. It can be seen that as the total number of users drops from seven to two (the number of users in each room may be different), the gesture recognition accuracy for new users drops from 0.9 to 0.79. This result is similar to the prediction result of BVP data new users in the literature [9], which can prove the validity of this work. The main reasons for the result are as follows: (1) Due to the different behavior habits of each user, the generated BVP data is in the distribution of users. There are differences: (2) The difference of user BVP can be compensated by the number of users; (3) The gesture recognition method based on BVP has environmental robustness.

**Figure 7:**
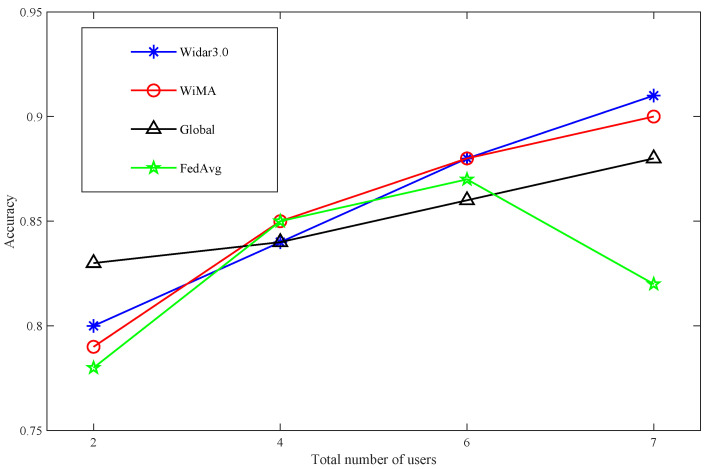
Comparison of WiMA algorithm with three methods—Room1.

**Figure 8:**
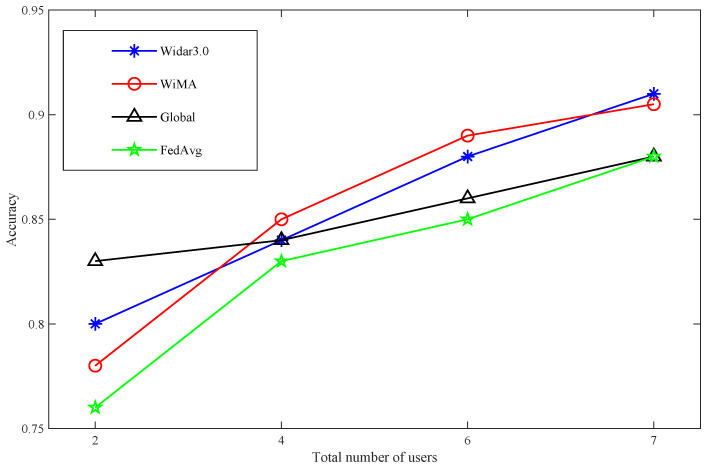
Comparison of WiMA algorithm with three method—Room2.

Comparison with other methods: The comparison of the accuracy of various methods with the number of users in different rooms is shown in Table 1; in order to show the results more intuitively, the same results are shown in pictures in Figure 7 and Figure 8. When the total number of users exceeds four, the accuracy of WIMA in both rooms can exceed 0.85, and when the number of users reaches seven, it reaches 0.9. Overall, WIMA can reach the standard of Widar 3.0. Its overall accuracy is better than that of Global and FedAvg, and, especially, the performance of FedAvg in room1 is much lower than that of WiMA and Widar3.0.

The main reasons are as follows: First, each neuron corresponds to a feature extractor, the arrangement positions of model neurons of different clients (rooms) are different, and the direct adoption of the overall average to update the model parameters may not be the optimal arrangement order of neurons. WiMA adopts the similarity matching of neurons layer by layer by freezing the model parameters, which can better capture the client model similarity characteristics [21]. Second, FedAvg is a simple average of client model parameters. If there is a distribution difference in the data of each client, it will extract common features as much as possible, which will affect the accuracy of some clients [29].

## 7. Conclusions

We proposed a gesture recognition system WiMA which exploited a federated matched averaging algorithm with WiFi signals. We focused on leveraging federated learning to address the accuracy and robustness of models with limited user data participating in model learning with different environments. Our experimental results illustrated that WiMA can improve the model accuracy where the data distribution is differentiated with two participant rooms. Although we initially implemented a gesture recognition solution for two rooms, our future work will continue to optimize the method and extend the results to more users and more scenarios.

## Figures and Tables

**Table 1:** Comparison of the accuracy of various methods with the number of users in different rooms.

	Room	Room1				Room2			
	User Num	2	4	6	7	2	4	6	7
Methods	Widar3.0	0.80	0.846	0.88	0.91	0.8	0.84	0.88	0.91
	WiMA	0.791	0.85	0.875	0.904	0.78	0.85	0.89	0.90
	Global	0.83	0.84	0.86	0.88	0.83	0.84	0.86	0.88
	FedAvg	0.78	0.85	0.87	0.82	0.76	0.83	0.85	0.88

## Data Availability

All data used in this study come from the open source dataset Widar3.0 of Tsinghua University, the link is http://tns.thss.tsinghua.edu.cn/widar3.0/.
